# *In Vitro* Characterization of Probiotic Strains *Bacillus subtilis* and *Enterococcus durans* and Their Effect on Broiler Chicken Performance and Immune Response During *Salmonella Enteritidis* Infection

**DOI:** 10.3390/microorganisms13020217

**Published:** 2025-01-21

**Authors:** Revathi Shanmugasundaram, Nalisa Khochamit, Ramesh K. Selvaraj, Mohammad Mortada, Surasak Siripornadulsil, Wilailak Siripornadulsil

**Affiliations:** 1 Toxicology and Mycotoxin Research Unit, U.S. National Poultry Research Center, Agricultural Research Service, U.S. Department of Agriculture, Athens, GA 30605, USA; 2 Department of Microbiology, Faculty of Science, Khon Kaen University, Khon Kaen 40002, Thailand; 3 Department of Poultry Science, The University of Georgia, Athens, GA 30602, USA

**Keywords:** *Salmonella Enteritidis*, *Bacillus subtilis*, *Enterococcus durans*, probiotics, broilers

## Abstract

*In vitro* experiments were conducted to characterize the effect of bile salt supplementation and pH on the proliferation of *Bacillus subtilis* CE330 and *Enterococcus durans* CH33 probiotics and *in vivo* experiments on production performance, cecal *Salmonella enterica* serovar Enteritidis (*S. Enteritidis*) load, and the immune response of broilers. A one-way ANOVA was used to examine the effect of bile and pH on probiotic species proliferation. *B. subtilis*. CE330 was more tolerant to high bile concentrations and pH levels compared to *E. durans* CH33. Bile concentrations between 3.0 and 4.0% and a pH range between 2 and 4 decreased (*p* < 0.05) the proliferation of *E. durans* CH33. *In vitro*, cell-free supernatants (CFSs) of *B. subtilis* CE330 and *E. durans* CH33 at a ratio of 1:1 significantly (*p* < 0.05) reduced *S. Enteritidis* proliferation, with the highest inhibition observed at a 5:1 ratio of *E. durans* CH33 CFS. The cultures of *B. subtilis* CE330 and *E. durans* CH33 with 4% bile salt for 72 h had a higher proline concentration of 56.95 (13.1-fold) and 20.09 (2.5-fold) µmol/g of fresh weight, respectively. A total of 144 one-day-old male Cobb broiler chicks were randomly allocated to four treatment groups—basal diet, basal diet + challenge, probiotics (*B. subtilis* CE330 and *E. durans* CH33, 0.5 g/kg feed), and probiotics + challenge in six replications. On day 14, birds in the challenge treatment were orally challenged with 1 × 10^8^ CFU of *S. Enteritidis*. A two-way ANOVA was used to examine the effects of probiotic supplementation and *Salmonella* challenge on dependent variables after 10 d post-*Salmonella* infection. Probiotic supplementation did not alter the body weight gain, the feed conversion ratio, the intestinal histomorphology (*p* > 0.05), or IL-1β and IL-10 gene expression (*p* > 0.05) at 10 dpi. However, probiotic supplementation decreased the *Salmonella* load by 38% compared to the control group. In conclusion, *B. subtilis* CE330 and *E. durans* CH33 reduced cecal *S. Enteritidis* load by 38%, thereby demonstrating their potential as probiotic interventions to enhance food safety and serve as alternatives to antibiotics in poultry. Hence, when developing multi-strain probiotic formulations, it is essential to emphasize the biocompatibility of various strains within the host system.

## 1. Introduction

*Salmonella* is a leading cause of foodborne illnesses globally, with significant implications for poultry production and public health [1]. In the United States and Europe, salmonellosis in humans is primarily linked with the consumption of *Salmonella* spp.-contaminated meat and eggs and poses a significant global public health concern [2]. In Asia, salmonellosis is often caused by contaminated poultry, seafood, and fresh produce, with *S. Typhimurium* and *S. Enteritidis* remaining predominant due to regional food safety challenges. While *Campylobacter jejuni* is a leading cause of foodborne illness globally, particularly in the United States and Europe, *Salmonella* remains more prevalent in terms of reported outbreaks associated with poultry and eggs [3]. The Centers for Disease Control and Prevention (CDC) estimates that *Salmonella* causes approximately 1.35 million infections in the United States annually [3]. Within the chicken house environment, *Salmonella* spp., particularly *Salmonella enterica* serovars Enteritidis (*S. Enteritidis*) and *S. Typhimurium*, are ubiquitous and represent significant foodborne pathogens of concern to the poultry industry [4,5]. These serovars are frequently identified in poultry flocks as early as 3 days of age, with the chicken intestinal tract serving as a primary reservoir for both *S. Enteritidis* and *S. Typhimurium* [6]. Despite *Salmonella* infection causing severe symptoms in humans, chickens infected with *S*. *enterica* often remain asymptomatic due to a downregulated inflammatory response facilitating the bacteria’s survival and persistence in the poultry gut for up to 10 weeks of age [7]. Furthermore, *Salmonella* infection leads to low levels of mucosal IgA and a gut-associated T-cell response in chickens; as a result, the T-cell and B-cell immune response does not protect the birds from *Salmonella* infections [8]. *Salmonella* colonization in the intestine plays a critical role in carcass contamination at slaughter, emphasizing the potential of reducing *Salmonella* colonization in chickens to decrease salmonellosis incidence in humans [9].

Regulatory programs in the European Union [10] and the National Poultry Improvement Plan (NPIP) [11] in the United States implemented stringent measures to control *Salmonella*, including mandatory monitoring and vaccination, targeting serovars like *S. Enteritidis* and *S. Typhimurium*, in commercial and breeders flocks to mitigate vertical transmission. Furthermore, the FDA Egg Safety Rule mandates routine testing for *S. Enteritidis* in commercial flocks to enhance food safety and reduce contamination risks [12]. Current control measures in poultry production have been effective to varying degrees but have limitations due to emerging antimicrobial resistance and regulatory restrictions on antibiotic growth promoters (AGPs) [13]. The emergence of multidrug-resistant strains linked to the inappropriate use of antibiotics in animal feed has become a significant global concern for consumers [14]. Despite efforts, on-farm control strategies, including vaccination, have had limited success in mitigating *Salmonella* contamination in chickens. Hence, alternative on-farm strategies to control *Salmonella* infection in broilers are critical. This has led to increased interest in alternative strategies, such as probiotics, for controlling *Salmonella* colonization in poultry [15,16]. Probiotics, or live microorganisms, contribute to maintaining gut health through the production of antimicrobial components and competitive exclusion, thereby maintaining gut homeostasis and protecting the host from pathogen infection [17,18].

Among the probiotic candidates, members of the Lactic Acid Bacterial (LAB) group, which includes *Bifidobacteria*, *Enterococcus*, *Lactobacillus*, *Pediococcus*, and *Streptococcus* [16], are well known for their ability to metabolize lactose into lactic acid, which lowers the gastrointestinal pH [19]. This acidification of the gastrointestinal tract creates an environment that is less conducive to the survival and growth of pathogens like *Salmonella*, which typically thrive more at a neutral pH [20]. Probiotics, including LAB and *Bacillus* microbial groups, can competitively exclude pathogenic bacteria like *Salmonella* by occupying binding sites on the intestinal epithelium [21]. This prevents the adhesion and colonization of *Salmonella*, reducing the likelihood of infection. Furthermore, probiotics play a role in modulating the composition and balance of the gut microbiota. A well-balanced and diverse gut microbiota can contribute to a healthier gut environment, making it less conducive to *Salmonella* colonization and infection [22]. *Bacillus subtilis*, in particular, has demonstrated that it can modify the intestinal microbiota, promoting the growth of LAB that are well known for their beneficial effects on intestinal health [23,24,25].

Due to its ease of production and prolonged shelf life, *B. subtilis* serves as a potential probiotic candidate and can enhance broiler performance [24,26], modulate immune responses [27], and maintain gut homeostasis during bacterial diseases. Additionally, *Enterococcus* spp., the third-biggest genus in the LAB group, demonstrates a wide temperature tolerance and is commonly used in the food industry as starter cultures, cocultures, or protective cultures [28]. Studies have demonstrated the potential of *E. durans* as a probiotic candidate, alleviating colitis through the induction of T regulatory cells and restoring gut microbial diversity in mice [29]. A panel of *Enterococcus* spp. (NLP-1319, 20, 24, 27, 34) was obtained from the duodenum, jejunum, ileum, cecum, and colon of the desi chicken (native) and showed great bile, acid, and phenol survival [20]. Furthermore, synbiotics made out of this autochthonous *E. durans* strain and microbial polysaccharides improve broiler chicken production performance. However, probiotic interactions within the chicken’s gastrointestinal tract ecosystem vary based on the probiotic strains and host animals [30]. With the emerging trend of using multi-strain probiotics as the norm for novel probiotic supplementation, increasing demand for exploring additional bacterial strains may contribute to novel and effective strategies for enhancing chicken health and performance through probiotic supplementation. The exploration of new bacterial strains could lead to the development of more tailored and efficient probiotic formulations for poultry applications. Additionally, limited studies have compared the effectiveness of probiotics against different *Salmonella* serovars in poultry production settings [31,32]. *E. durans* is emerging as a promising probiotic candidate, along with other probiotic strains like *Bacillus.* Hence, this study aimed to investigate the effects of a combination of *B. subtilis* CE330 and *E. durans* CH33 supplementation on the growth performance and immune response of chickens 10 d post-*S. Enteritidis* infection. An *in vitro* study was also conducted to evaluate the effects of bile salt supplementation and pH on the proliferation of *B. subtilis* CE330 and *E. durans* CH33, thereby suggesting their potential as an alternative to antibiotics.

## 2. Materials and Methods

The animal use protocol was approved by the Institutional Animal Care and Use Committee at the University of Georgia (AUP: A2018 06-026-R1).

### 2.1. Bacterial Strains and Culture Conditions

*B. subtilis* CE330 (MN848256) isolated from the cecum of 30-day-old broilers [33] and *E. durans* CH33 (MF066896.1) isolated from the intestine of domestic fowl [34] were used as probiotics in both *in vitro* and *in vivo* experiments. The 16S DNA sequences of *B. subtilis* CE330 (MN848256) and *E. durans* CH33 (MF066896.1) are given in the Supplementary Materials. Wild-type *S. Enteritidis* was used in *Salmonella* challenge studies [35].

### 2.2. In Vitro Characterization of Probiotic Strains

#### 2.2.1. pH and Bile Salts on Probiotic Proliferation

Probiotic strains *B. subtilis* CE330 and *E. durans* CH33 were isolated from a single colony and isolated in Luria–Bertani (LB) broth at 37 °C for 24 h with continuous shaking at 150 rpm. Subsequently, overnight probiotic cultures were transferred to 50 mL of deMan, Rogosa, and Sharpe (MRS) (Sigma Aldrich, St. Louis, MO, USA) broth and incubated at 37 °C for 48 h. The probiotic culture’s optical density (OD) was measured at a 600 nm wavelength using a spectrophotometer (BioTek, Winooski, VT, USA). The probiotic cultures were incubated until they reached the OD value between 0.9 and 1.2.

For the bile salt tolerance test, 100 μL of overnight probiotic cultures were resuspended in 5 mL of LB or MRS broth (1 × 10^5^ colony-forming units (CFUs) *B. subtilis* CE330 or 1 × 10^9^ CFU *E. durans* CH33) supplemented with 0, 2.5, 3.0, 3.5, or 4.0% *bile* salts [36] (Sigma-Aldrich, St. Louis, MO, USA) and incubated at 37 °C for 4 h. (*n* = 3).

For the acid pH tolerance test, 1 × 10^5^ CFU *B. subtilis* CE330 or 1 × 10^9^ CFU *E. durans* CH33 were incubated at 37 °C for 4 h in LB or MRS broth with the pH adjusted to 2, 3, 4, 5, 6, or 7 by using 1 N HCl or 1 N NaOH. LB or MRS broth at pH 7 or 6. 4, with no pH adjustment, was used for the control treatment. After 4 h of incubation, the OD_600_ was measured using a spectrophotometer to assess the probiotics’ survivability in the culture (*n* = 3).

All the *in vitro* assays were replicated in triplicates across three independent experiments (*n* = 3).

#### 2.2.2. Cell-Free Supernatant of *B. subtilis* CE330 and *E. durans* CH33 on *S. Enteritidis* Proliferation *In Vitro*

The cell-free supernatant (CFS) of a single probiotic strain, either *B. subtilis* CE330 or *E. durans* CH33, was obtained by centrifuging probiotic cultures at 3075× *g* at 4 °C for 15 min upon reaching an OD value between 0.9 and 1.2. Subsequently, the CFS was further filtered using a 0.22 µm filter (EMD Millipore, MA, USA). A 10 µL overnight culture of *S. Enteritidis at* 1 × 10^6^ CFU/mL was co-incubated with the CFS of each probiotic strain at 0:1, 1:1, 5:1, and 10:1 ratios to the pathogen conducted in triplicate (*n* = 3) in 96-well plates, and the total volume was adjusted to 110 µL per well. Plates were incubated at 37 °C for 24 h. After 24 h of incubation, OD_600_ was measured using a spectrophotometer (BioTek, VT, USA) to evaluate the inhibitory effect of probiotic culture supernatant on *Salmonella* proliferation [37].

#### 2.2.3. Determination of the Proline Content in *B. subtilis* CE330 and *E. durans* CH33

In chickens, the pH in the gut ranges from 1.2 to 6.8 [38], and the *in vivo* supplementation of these bacterial strains aims to enhance their survival under varying gastric pH conditions. Proline is known for its accumulation in microorganisms and is accountable for a variety of stress factors, such as biotic interactions like pathogen attacks or symbiotic relationships, and abiotic stress, such as osmotic stress. Measuring proline levels serves as a valuable indicator, providing insights into the stress tolerance and adaptation of microorganisms within the host. To assess the stress tolerance of probiotic cultures *B. subtilis* CE330 and *E. durans* CH33 under different pH and bile salt concentrations, a ninhydrin test was used to quantify proline concentration. The probiotics were cultured without bile salt (0%) and at pH 7.0 for *B. subtilis* CE330 and 6.4 for *E. durans* CH33 for 24 h. Bacterial cells were also cultured at pH 2 to 5 and with 1% to 4% bile salt concentrations, respectively. Proline content in the samples was measured at 24 h, 48 h, and 72 h of culture, as described by Chinard et al. [39]. Briefly, a 100 mg bacterial cell pellet was homogenized in either 1 or 5 mL of 3% (*w*/*v*) 5′-sulfosalicylic acid and then centrifuged at 3075× *g* and 4 °C for 10 min. Then, 1 mL of supernatant was combined with 1 mL of ninhydrin reagent, and the mixture was heated at 100 °C for 1 h. The reaction was placed in an ice bath, and two mL of toluene solution was added to stop the activity. The absorbance was measured at 520 nm, and the proline content was expressed as µmol per g of fresh-weight cells. Proline concentration was determined from a proline standard curve.

### 2.3. In Vivo S. Enteritidis Challenge Study

The *in vivo* study aimed to investigate whether the supplementation of probiotic strains *B. subtilis* CE330 and *E. durans* CH33 could provide protection to broiler chickens against *S. Enteritidis* infection.

#### 2.3.1. Preparation of Probiotics

A 5% (*v*/*v*) dilution of 18 h and 48 h cultures of *B. subtilis* CE330 and *E. durans* CH33 was transferred to 2 L of modified LB broth containing 2% soybean meal, 0.5% maltose, 0.05% KH_2_PO_4_, and 0.1% MnSO_4_ at pH 7.5. The mixture was then incubated at 37 °C for 7–14 d to induce spore germination. Following incubation, the cultures were centrifuged at 3075× *g* and 4 °C for 15 min to obtain the soybean meal pellets containing bacterial cells. The pellets were subsequently dried using a hot air oven at 50 °C for 7 to 10 d until a constant dried weight was achieved. The dried samples were stored at 4 °C until further analysis. Prior to use in the experiment, the viable cell counts of each bacterial strain were determined both before and after the drying process to ensure the bacterial strains’ viability. Briefly, one gram of probiotic powder was dissolved in 9 mL of sterile peptone water. Subsequently, cell counting was conducted through serial dilutions starting from the initial suspension, followed by plating on LB agar for *B. subtilis* CE330 and on MRS agar for *E. durans* CH33. The agar plates were then incubated at 37 °C for 48 h. The viable cells were quantified and expressed as colony-forming units per gram (CFU g^−1^).

#### 2.3.2. Birds and Housing

A total of 144 one-day-old male Cobb-500 broiler chicks (Cobb-Vantress hatchery, Cleveland, GA, USA) were housed in four Petersime battery brooder cages (70 cm × 60 cm × 30 cm) in a thermostatically controlled room at the animal biosafety level-2 research facility. The lighting and temperature conditions were controlled in accordance with the Cobb Broiler Management Guide [40]. Chicks were randomly allocated to one of the four treatment groups: basal diet (corn-soybean meal diet), basal diet + *S. Enteritidis* challenge, probiotics (*B. subtilis* CE330 and *E. durans* CH33, 0.5 g/kg feed), and probiotics + *S. Enteritidis* challenge. The birds were fed probiotics from d1. Each treatment was replicated in six battery cages (*n* = 6), with six birds per pen. The composition of the basal diet is provided in Table 1. *B. subtilis* CE330 and *E. durans* CH33 were mixed at a ratio of 1:1 prior to addition to the basal diet at a final concentration of 0.05% (*w*/*w*). The birds had ad libitum access to feed and water.

The wild-type strain of *S. Enteritidis* was cultivated on plates containing XLT-4 agar for a duration of 48 h at 37.5 °C. Subsequently, bacterial cells were inoculated into a brain heart infusion broth and allowed to incubate at 37.5 °C without shaking for an additional 48 h. Following this incubation period, bacterial cells were collected by centrifugation at 3500× *g* at 4 °C and then resuspended in PBS to achieve a concentration of 1 × 10^8^ colony-forming units (CFUs) per mL. The accuracy of the challenge dose was verified by plating serial dilutions on XLT-4 agar. No *Salmonella* was detected in cloacal swab samples collected from the birds before they were assigned to their respective treatments and before infection at 14 days of age. On day 14, the broilers in the challenge treatment groups were orally gavaged with 1 × 10^8^ CFUs of *S. Enteritidis*. Body weight and feed consumption were recorded weekly to determine the body weight gain (BWG) and feed conversion ratio (FCR). The cytokine gene expression, jejunal histological morphology, cecal *B. subtilis* CE330, *E. durans* CH33 loads, and *S. Enteritidis* load were measured at 10 d post-infection (dpi) based on a previous study [41].

#### 2.3.3. Histological Morphology of the Jejunum

At 10 dpi, 2 cm sections of jejunal samples proximal to Meckel’s diverticulum were collected from each pen (*n* = 6) for histomorphology analysis. The jejunum tissue samples were stored in 10 mL of buffered formalin until further processing. Histo-morphological analysis was conducted in accordance with the methods outlined in Shanmugasundaram et al. [35]. Briefly, the fixed samples were dehydrated in a graded ethanol series (50%, 70%, and 96% for 15 min each and absolute ethanol for 30 min, with one change at 15 min) prior to embedding in paraffin using a Leica TP 1020 tissue 45 processor (GMI Inc., Ramsey, MN, USA). The embedded samples were sectioned into 5 μm thick sections, mounted on slides, and then stained with hematoxylin and eosin. Cross-sections of jejunum samples were examined using CellSens Imaging software, version 4.3.1 (Olympus America, Central Valley, PA, USA). The villus height and crypt depth were measured in five sections of each tissue sample for at least five fields per section.

#### 2.3.4. Bacterial Quantification in Cecum

At 10 dpi, bacterial genomic DNA was isolated from the cecal content, as described earlier by Shanmugasundaram et al. [35]. Extracted genomic DNA was purified by using DNA purification columns and washed twice with ice-cold 75% ethanol. Purified genomic DNA was eluted and resuspended in TE buffer (10 mM Tris-HCl, 1 mM EDTA, pH 8.0) and maintained at −20 °C. The purified genomic DNA from the cecal content and the total number of bacteria were quantified by qPCR [42]. Three sets of primers were designed for *B. subtilis* and *E. durans* (16S DNA sequences of *E. durans* CH33 and *B. subtilis* CE330 are provided in the Supplementary Materials) and evaluated in silico. The corresponding primers were blasted against each respective bacterial strain. The results of the PCR amplification products of *B. subtilis* and *E. durans* were examined using gel electrophoresis on 1% agarose gel to verify that a single product was generated at the predicted size. Single-band PCR products for each gene were excised and purified with a QIAquick Gel Extraction Kit (Qiagen, Valencia, CA, USA) and subsequently sequenced at the Georgia Genomics and Bioinformatics Core (University of Georgia, Athens, GA, USA). The genomic sequences of *B. subtilis* and *E. durans* were used to design primers and amplify a PCR product that had a maximum identity to its corresponding gene of above 99% (Standard nucleotide–nucleotide BLAST, NCBI). The specific primer pairs used to quantify *B. subtilis*, *E. durans*, and *S. Enteritidis* are shown in Table 2. The quantification cycle (C_q_*)* values were determined using iQ5 software (Bio-Rad, Hercules, CA, USA). For absolute quantification, the C_q_ value was compared to the standard curve generated for each bacterial species, as described earlier [37]. The PCR amplification efficiencies for *B. subtilis*, *E. durans*, and *S. Enteritidis* were 83.0, 96.1, and 102.1%, respectively. The slope and intercept of the standard curves for *B. subtilis*, *E. durans*, and *S. Enteritidis* were (1) C_q_ = 3.811 × Log (DNA) + 20.724, (2) C_q_ = 3.419 × Log (DNA) + 26.339, and (3) C_q_ = 3.272 × Log (DNA) + 10.491, respectively. The copy number of each bacterial species was calculated using the formula as described previously [37].

#### 2.3.5. Cecal Tonsil Cytokine Expression

Cecal tonsils were collected from one bird per pen in RNAlater (Qiagen, Germantown, MD, USA) and stored at −80 °C until further analysis (*n* = 6). Total RNA was extracted and reverse-transcribed into cDNA, as described by Selvaraj et al. [46]. The relative expression of IL-10 and IL-1β mRNA levels was quantified by SYBR Green qPCR. The cytokines were chosen based on a previous study [47]. The primer sequences and the annealing temperatures are given in Table 2. Ribosomal protein S13 (RPS13) and glyceraldehyde-3-phosphate dehydrogenase (GAPDH) were used as internal controls for normalization of the target mRNA gene expression. Fold change from the control group was calculated using the 2^(−∆∆Ct)^ method, as described earlier [48].

## 3. Statistical Analyses

For the *in vitro* assays, one-way ANOVA (JMP Software, version 16.2.0, Cary, NC, USA) was used to examine the effect of the different parameters studied on dependent variables. For the *in vivo* study, a 2 × 2 factorial analysis was used to examine the effect of probiotic supplementation, *S. Enteritidis* challenge, and their interaction on the dependent variables. When the interaction effects were not significant (*p* < 0.05), the main effects were analyzed. Differences between means were analyzed by Tukey’s HSD test.

## 4. Results

### 4.1. Effect of pH and Bile Salts on Probiotic Proliferation

**Figure 1:**
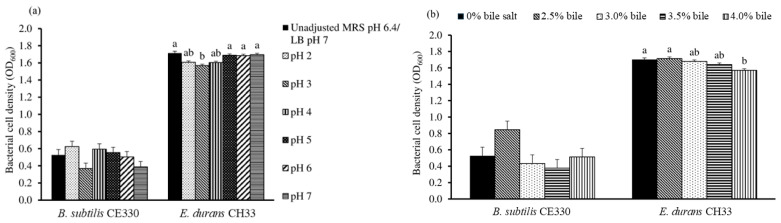
Effect of pH 2.0 to 7.0 (**a**) and a bile salt concentration of 2.5 to 4.0% (**b**) on the OD_600_ of *B. subtilis* CE330 and *E. durans* CH33 cultures after 4 h of exposure to acidic or bile-salt-containing conditions. The bars are the means ± SEMs. Bars with no common letters within the same bacterial species differ significantly at *p* < 0.05. The *p* values of the pH tolerance test of *B. subtilis* CE330 and *E. durans* CH33 were 0.253 and 0.002, respectively. The *p* values of the bile salt tolerance test of *B. subtilis* CE330 and *E. durans* CH33 were 0.131 and 0.016, respectively. (*n* = 3).

Altering the pH between 2.0 and 7.0 in the culture media did not significantly decrease the growth of *B. subtilis* CE330 and *E. durans* CH33, except at pH 3.0, at which *E. durans* CH33 had significantly (*p* < 0.05) decreased growth when compared to that in the control (Figure 1a). Bile salts between 2.5% and 4% did not decrease the growth of *B. subtilis* CE330 (Figure 1b). *E. durans* CH33 growth was significantly (*p* < 0.05) decreased when supplemented with 4% bile salts for 4 h.

### 4.2. Effect of B. subtilis CE330 and E. durans CH33 CFS on S. Enteritidis Proliferation In Vitro

**Figure 2:**
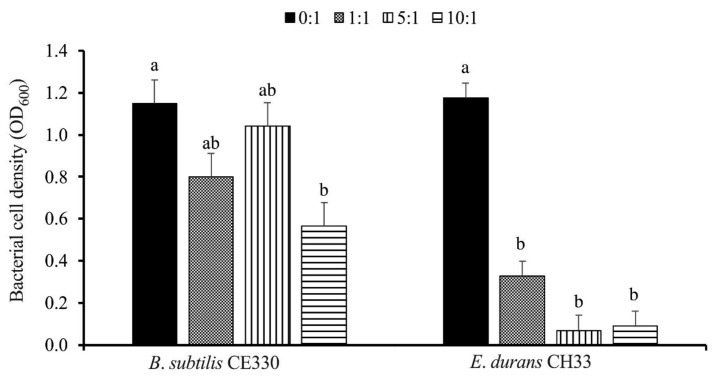
Effect of the cell-free supernatant (CFS) of *B. subtilis* CE330 and *E. durans* CH33 on *S. Enteritidis in vitro* proliferation (*n* = 3) after 24 h of incubation. A total of 10 µL of 1 × 10^6^ CFU/mL *S. Enteritidis* was incubated with the CFS of each strain at four different ratios between supernatant and pathogen (0:1, 1:1, 5:1, and 10:1). The bars are the means ± SEM; *n* = 3 per treatment group. Bars with no common letters within the same bacterial species differ significantly at *p* < 0.05. *p* values of the *in vitro* assay of the inhibition activity of *B. subtilis* CE330 and *E. durans* CH33 were 0.025 and <0.0001, respectively.

*B. subtilis* CE330 and *E. durans* CH33 CFS inhibited the proliferation of *S. Enteritidis* (Figure 2). The maximal inhibition of *S. Enteritidis* proliferation was observed with the *E. durans* CH33 CFS. Twenty-four hours of *in vitro* incubation of *E. durans* CH33 CFS with *S. Enteritidis* at ratios of 1:1, 5:1, and 10:1 significantly (*p* < 0.05) decreased the OD values from 1.17 to 0.33, 0.07, and 0.09, respectively, which correspond to approximately 72% to 94% reductions in *S. Enteritidis* proliferation. Similarly, 24 h of *in vitro* incubation of *B. subtilis* CE330 CFS with *S. Enteritidis* at ratios of 1:1, 5:1, and 10:1 decreased the OD value from 1.15 to 0.8, 1.04, and 0.57, respectively. At a ratio of 10:1 of *B. subtilis* CE330 CFS to *S. Enteritidis*, proliferation was significantly reduced compared with that in the 0:1 control group (Figure 2).

### 4.3. Effect of Low pH and Bile Salt Stress on Proline Content of B. subtilis CE330 and E. durans CH33

**Figure 3:**
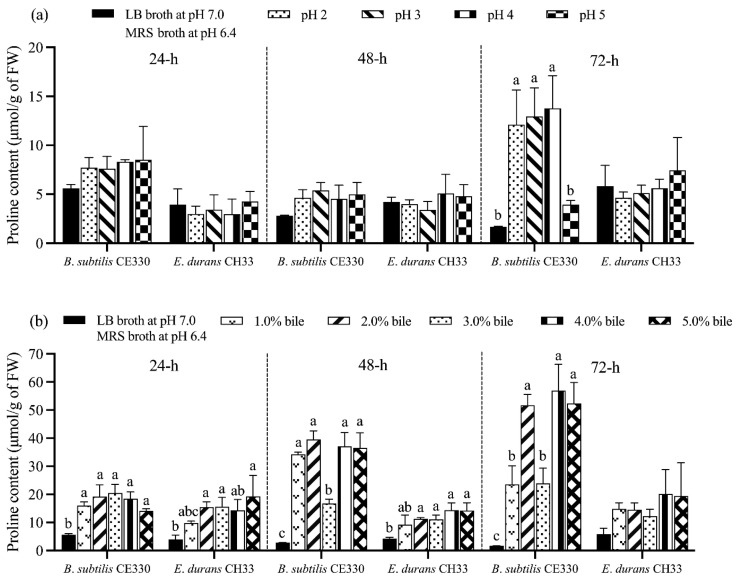
Effect of pH 2.0 to 5.0 (**a**) and a bile salt concentration of 1.0 to 5.0% (**b**) on the proline content of *B. subtilis* CE330 and *E. durans* CH33 cultures during a 24 h to 72 h period. The bars are means ± SEM; *n* = 3 per treatment group. Bars with no common letters within the same bacterial species differ significantly at *p* < 0.05. The *p* values of the proline concentration of CE330/CH33 under various pH conditions at 24 h, 48 h, and 72 h of culture were 0.310/0.074, 0.070/0.428, and <0.001/0.460, respectively. The *p* values of the proline production of CE330 under various bile salt concentrations at 24 h, 48 h, and 72 h of culture were <0.001, <0.001, and <0.001, respectively. The *p* values of the proline concentration of CH330/CH33 under various bile salt concentrations at 24 h, 48 h, and 72 h of culture were 0.001/0.005, <0.001/0.001, and <0.001/0.137, respectively.

Incubation with *B. subtilis* CE330 at pH levels ranging from 2 to 5 for 72 h resulted in a significant reduction in proline concentrations, decreasing from 12.10 to 3.93 μmol/g of bacteria (*p* < 0.001). By contrast, *E. durans* CH33 showed no significant change in proline concentration under similar conditions during the same period (*p* = 0.460) (Figure 3a).

When *B. subtilis* CE330 was incubated with 2% bile salt at pH 7, the proline concentration significantly increased from 19.23 μmol/g at 24 h to 39.61 μmol/g of bacteria at 48 h (*p* < 0.001). Furthermore, after 72 h of incubation with 4% bile salt, the highest proline concentration was recorded for both strains. At 72 h, *B. subtilis* CE330 reached a proline concentration of 56.95 μmol/g (*p* < 0.001), while *E. durans* CH33 reached a proline concentration of 20.09 μmol/g of bacteria (*p* = 0.137) (Figure 3b).

### 4.4. Effect of Probiotic Supplementation on Performance Parameters During S. Enteritidis Challenge

At 10 dpi, there were no significant (*p* > 0.05) main and interaction effects of treatment on BWG and FCR (Table 3).

### 4.5. Effect of Probiotic Supplementation on Intestinal Histomorphology During S. Enteritidis Challenge

**Figure 4:**
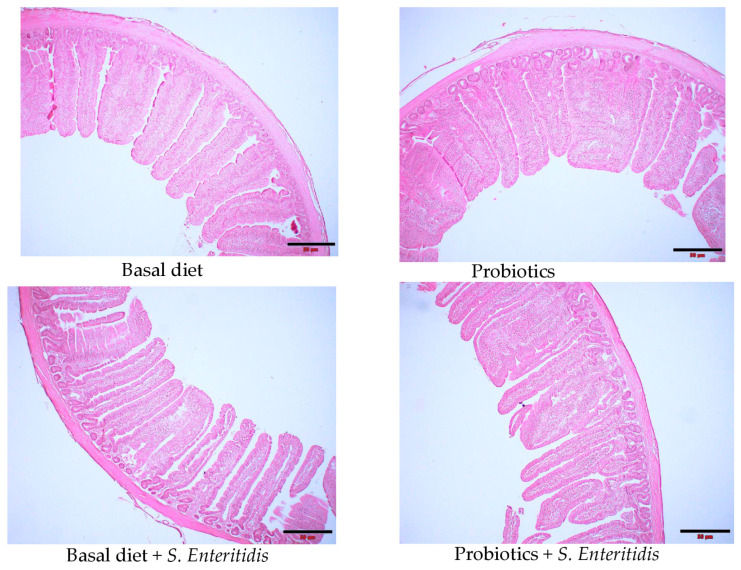
Effect of dietary supplementation with the *B. subtilis* CE330 and *E. durans* CH33 probiotic mixture on jejunal histomorphology of broiler chickens on d 24. (Scale bar 50 µm).

At 10 dpi, there were no significant (*p* > 0.05) main or interaction effects of treatment on jejunal villus height, jejunal crypt depth, or the villus height/crypt depth (Table 4). However, the probiotic-supplemented group had a trend of increased jejunal villus height compared with the control group (*p* > 0.05). (Figure 4).

### 4.6. Effect of Probiotic Supplementation on Cecal S. Enteritidis Load

**Figure 5:**
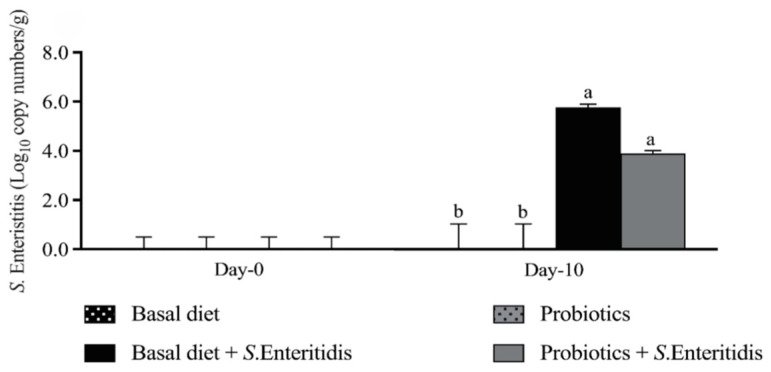
Effect of dietary supplementation with the *B. subtilis* CE330 and *E. durans* CH33 probiotic mixture on the concentration (Log_10_ copy number/g) of *S. Enteritidis*, in the ceca of 24 d old male broilers from both the nonchallenged group and *S. Enteritidis*-challenged group. The bars are means ± SEMs; *n* = 6 per treatment group. Bars with no common letters differ significantly at *p* < 0.05. Effect of probiotic supplementation and *S. Enteritidis* challenge on the cecal *S. Enteritidis* load. *p* value of treatment challenge = 0.3007, challenge = <0.0001, and treatment = 0.3773.

At 10 dpi, the cecal *S. Enteritidis* load in the challenged groups was higher (*p* < 0.001) than that of the chicks in the nonchallenged groups (Figure 5). The *S. Enteritidis* load in the control unchallenged group was below detectable levels at ~1.5 log_10_. The combined *B. subtilis* CE330 and *E. durans* species supplementation decreased the *Salmonella* load by 2.4 logs_10_ compared to the control challenge group (*p* < 0.05) (Figure 5).

### 4.7. Effect of Probiotic Supplementation on Cecal Tonsil Immune Gene Expression

**Figure 6:**
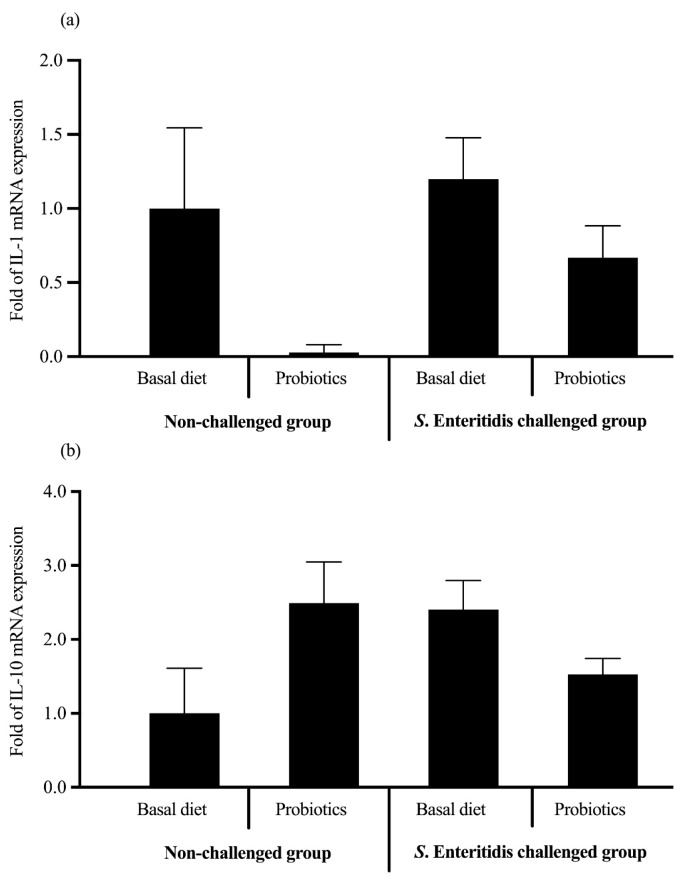
Effect of supplementation with the *B. subtilis* CE330 and *E. durans* CH33 probiotic mixture on the expression of (**a**) cecal tonsil IL-1β and (**b**) cecal tonsil IL-10 levels in 24 d old male broilers from both the nonchallenged group and *S. Enteritidis*-challenged group. The bars are means ± SEMs; *n* = 6 per treatment group. Two-way ANOVA of the results of the *in vivo* assay of the effect of probiotic supplementation and *S. Enteritidis* challenge on cecal tonsil IL-1β and IL-10 gene expression revealed no significant interaction or main effects (IL-1β: *p* value of treatment × challenge = 0.860, *p* value of challenge = 0.171, *p* value of treatment = 0.150; and IL-10: *p* value of treatment × challenge = 0.501, *p* value of challenge = 0.972, *p* value of treatment = 0.927, respectively).

There were no significant (*p* > 0.05) main and interaction effects of treatment on IL-1 (Figure 6a) and IL-10 mRNA levels (*p* > 0.05) (Figure 6b).

## 5. Discussion

*B. subtilis* CE330 and *E. durans* CH33, isolated from the GIT of broilers, were evaluated for their tolerance to acid and bile salt, antimicrobial activity, impact on broiler performance, cecal bacterial loads, and immune response during *S. Enteritidis* infection. The isolated *B. subtilis* CE330 and *E. durans* CH33 demonstrated resistance to acid and bile salt conditions, successfully colonized the GIT, and inhibited *S. Enteritidis* proliferation *in vitro*. Incubating *B. subtilis* CE330 and *E. durans* CH33 with 4% bile salt increased the proline content of the probiotic strains. Our *in vitro* findings suggest their potential as promising probiotics for broiler chickens to control *Salmonella* in the chicken gut, consistent with previous reports by [33,34].

Both *B. subtilis* CE330 and *E. durans* CH33 exhibited resistance to low pH (2.0) and high bile salt concentrations (3.5–4.0%) *in vitro*. pH and bile salt tolerance are critical for not only colonization but also survival in the GIT [49]. The hydrogen ion or proton pump system in bacteria is responsible for acid tolerance [50]. The presence of the bile salt hydroxylase (BSH) enzyme in *B. subtilis* CE330 and *E. durans* CH33 can be expected to contribute to their resistance to bile salts, reducing the toxic effects of bile salts [51,52], which can facilitate gut colonization in chickens.

Antimicrobial activity is one of the important properties of potential probiotics, which can improve competitive ability by inhibiting the growth of other bacteria, especially enteric pathogens. The CFS from *B. subtilis* CE330 and *E. durans* CH33 showed inhibitory effects, suggesting their potential antimicrobial activity against *S. Enteritidis*. The antimicrobial substances produced by *B. subtilis* CE330 and *E. durans* CH33, such as bacteriocins and bacteriocin-like inhibitory substances, most likely play a role in inhibiting the growth of *S. Enteritidis*. Various species of *Bacillus* and *Enterococcus*, including *B. subtilis* and *E. durans*, are capable of producing a number of bacteriocins that inhibit *Salmonella* species [34,53,54,55,56]. However, the antimicrobial activity of CFSs from probiotic strains can be specific to the species and serovars of pathogenic bacteria. Studies showed that *E. durans* LAB18s CFS did not exert antimicrobial activity against *S. Enteritidis*, though its intracellular extract exhibited inhibitory activity against *S. Enteritidis* [53]. This finding suggests that the antimicrobial activity of the CFS from probiotic strains is species-specific and serovar-specific to the pathogenic bacteria [57]. Furthermore, *B. subtilis* CE330 and *E. durans* CH33 CFS have inhibitory effects on *S. Enteritidis*, which suggests that these strains could be used as a preventive measure to reduce the incidence of *Salmonella* infections in chickens.

Proline is recognized as a significant source of carbon and nitrogen for diverse bacteria, protecting against oxidative stress. This protective function is achieved through maintaining intracellular redox homeostasis and elevating catalase activity [58]. *B. subtilis* CE330 and *E. durans* CH33 increasing the synthesis of proline under acidic environmental conditions suggests that proline contributes to counteracting acid-stress-induced osmotic stress in bacteria. The secretion of substantial proline amounts by these probiotic strains may provide significant advantages for colonizing the chicken gut. These benefits include a diverse aspect, including survival and colonization in acidic gastric environmental conditions in the host, growth, immunity, and gut health [59,60]. Future studies might further explore the complex mechanisms through which proline contributes to stress resilience in probiotics.

Several studies have shown that supplementing probiotics with broiler feed can enhance growth performance in broiler production [61,62,63]. Studies demonstrated that administering either a single *B. subtilis* CE330 strain or a combination with four LAB strains had positive effects on broiler growth at 45 days of age [64]. However, in our present study, feeding a probiotic mixture comprising *B. subtilis* CE330 and *E. durans* CH33 had no significant effect on BWG and FCR until d 24. This suggests that short-term probiotic supplementation did not affect production performance. This finding aligns with the observations by Zhang et al. [65], who reported that *B. subtilis* UBT-MO2 administration had no significant difference in BWG at day 21 compared to control chicks. The biocompatibility and relationship between probiotic species and broiler growth performance are complex and influenced by several factors such as gut health, intestinal morphology, and microbiota composition [66,67,68].

There were no significant differences in villus height, crypt depth, or villus height/crypt depth ratio among treatment groups. The failure to improve growth performance and intestinal morphology may be due to the levels, routes, and combinations of probiotic supplementation [69,70]. Moreover, unlike monocomponent probiotics, whose focus is on a single strain, biocompatibility among diverse strains becomes a primary concern for multi-strain formulations. Developing multi-strain probiotics requires careful consideration of how various strains will interact within the host’s system [71].

In this study, *B. subtilis* CE330 and *E. durans* CH33 did not alter the cecal *B. subtilis* and *E. durans* load compared with that in the control basal-diet-fed chicks. This indicates that these probiotics need to be supplemented continuously. Studies with a collection of *Enterococcus* spp. strains (NPL1315-NPL1334) were obtained from various segments of the digestive tract, including the duodenum, jejunum, ileum, caecum, and colon, of desi chickens (native breed) [20]. Specifically, chosen strains (NLP-1319, 20, 24, 27, 34) exhibited favorable resistance to bile, acid, and phenol, as well as cell surface hydrophobicity. Subsequently, an *in vivo* trial was conducted on young male Ross chickens to assess the efficacy of a custom synbiotic formulation. This formulation consisted of an *E. durans* strain (1 × 10^8^ CFUg^−1^) combined with prebiotics Dextran or Levan (0.1% *w*/*v*), either individually or in combination, which significantly increased the body weight, along with reduced levels of serum cholesterol, triglycerides, and glucose. Additionally, a decreased pathogenic load was observed in the synbiotic-fed chickens [20,53,68]. These findings suggest that the *E. durans* strain isolated from native chicks effectively functions as a probiotic in broilers [20]. However, the complexity and dynamics of microbial composition can be influenced by several factors, including probiotic administration, genetics, diet, housing, and environment [72,73]. Supplementation of *B. subtilis* CE330 and *E. durans* CH33 decreased the *Salmonella* load by 2.4 logs 10 d post-challenge. It is important to highlight that the multi-strain probiotics were administered through feed starting from day 1, with the *Salmonella* challenge on day 14. Furthermore, sample collection occurred 10 days post-infection (dpi). The *in vitro* assays demonstrated that the CFS of *B. subtilis* CE330 and *E. durans* CH33 significantly inhibited *S. Enteritidis* proliferation. Probiotics can enhance gut health by producing antimicrobial compounds, such as bacteriocins and short-chain fatty acids (SCFAs), and by promoting the competitive exclusion of pathogens [74]. While this study primarily focused on the antimicrobial effects of *B. subtilis* CE330 and *E. durans* CH33 against *S. Enteritidis*, these mechanisms are likely to play a role in *Salmonella* reduction [75]. This finding suggests that these probiotics produce antimicrobial compounds, such as bacteriocins and short-chain fatty acids (SCFAs), which lower gut pH and limit the ability of *Salmonella* to colonize and persist in the gut environment [75]. However, this study did not directly investigate microbiota composition or SCFA production. Furthermore, the observed reduction in cecal *Salmonella* load by 2.4 logs *in vivo* may be associated with competitive exclusion, where probiotics occupy intestinal binding sites, which may prevent *Salmonella* adhesion and colonization [76,77]. The reduction in cecal *Salmonella* load observed in this study suggests that *B. subtilis* CE330 and *E. durans* CH33 might have modulated the gut microbiota environment to favor beneficial microbes, thus inhibiting pathogen persistence. The antimicrobial effects observed in our study are likely multifactorial, involving both direct inhibition through antimicrobial compound production and indirect suppression via competitive exclusion. These mechanisms may work synergistically to reduce *Salmonella* colonization in the gut; this study did not directly quantify the relative contributions of antimicrobial compound production versus competitive exclusion [78,79].

Probiotics have been reported to enhance the host’s immune response. By modulating the cytokine response, probiotics may help the host organism defend against *Salmonella* and other infections more effectively [80]. Even though there was no significant difference in the expression of the IL-1β and IL-10 genes, there was a decreasing trend in the expression of IL-1β in the probiotic-fed broilers compared to the control chickens. These findings suggest that optimizing the dose of *B. subtilis* and *E. durans* in the feed may alleviate *S. Enteritidis*-induced inflammation in the broiler intestine.

Our findings suggest that antimicrobial compound production by *B. subtilis* CE330 and *E. durans* CH33 plays a dominant role in inhibiting *S. Enteritidis* proliferation. The significant inhibition of *S. Enteritidis* proliferation (94%) observed *in vitro* with the cell-free supernatants at a 5:1 ratio emphasizes the potential of bacteriocins and SCFAs as key mechanisms. While competitive exclusions and gut microbiota modulation may also contribute, the direct antimicrobial activity appears to be the primary mechanism under the conditions studied. Future research should focus on identifying and characterizing the specific antimicrobial compounds produced by these probiotics, metagenomic sequencing [81], and metabolomic profiling [82] to investigate how *B. subtilis* CE330 and *E. durans* CH33 modulate the gut microbiota, including SCFA production and competitive interactions with pathogens. For instance, gas chromatography–mass spectrometry (GC-MS) or liquid chromatography–mass spectrometry (LC-MS) could be used to detect specific antimicrobial compounds, such as bacteriocins or short-chain fatty acids (SCFAs), and to map metabolic pathways involved in pathogen inhibition [83,84]. Furthermore, in ovo supplementation of *B. subtilis* CE330 and *E. durans* CH33 through the amniotic route or prior to hatching would complement the existing research by enabling precise tracking of metabolic interactions at the cellular and molecular level, further validating the observed effects *in vivo*.

## 6. Conclusions

This study demonstrated that *B. subtilis CE330* and *E. durans CH33*, which were originally isolated from the GIT of broiler chickens, have favorable characteristics as potential probiotics for broiler chickens, particularly in terms of acid and bile salt tolerance, and inhibit *S. Enteritidis* proliferation *in vitro*. The *in vitro* results indicate that the antimicrobial effects of these probiotics are primarily associated with the production of inhibitory compounds, as evidenced by the significant reduction in *S. Enteritidis* proliferation when exposed to the CFS of these strains. *B. subtilis* CE330 and *E. durans* CH33 supplementation did not show a significant impact on production performance; however, it decreased the cecal *S. Enteritidis* load by 2.4 log_10_ 10 d post-challenge. These findings suggest that the *B. subtilis* CE330 and *E. durans* CH33 may employ a combination of strategies—such as antimicrobial compound production and competitive exclusion—to inhibit *S. Enteritidis*. Future research should focus on distinguishing between these mechanisms through targeted in ovo supplementation of *B. subtilis* CE330 and *E. durans* CH33 experiments and adhesion assays to test competitive exclusion and metabolomic analyses to identify and quantify antimicrobial compounds. Such studies will help clarify the roles of direct pathogen inhibition versus microbiota modulation in reducing *S. Enteritidis* colonization. This study emphasizes the potential for using *B. subtilis* CE330 and *E. durans* CH33 as alternatives to antibiotics in poultry production, with implications for improving food safety and public health. However, further investigations are warranted to optimize dosing strategies, evaluate long-term effects, and better understand the interaction between these probiotics and the intestinal microbiota.

## Figures and Tables

**Table 1:** Basal diet ingredients and calculated nutrient composition.

	Starter (0–24 d)
Ingredients	%
Corn	58.48
Soybean meal, 48%	35.15
Soybean oil	2.27
Monocalcium phosphorus, 21%	1.38
Limestone	1.59
DL-Methionine	0.21
L-Lysine-HCL, 78%	0.14
Salt (NaCl)	0.35
Vitamin premix ^1^	0.08
Mineral premix ^2^	0.35
Total	100.0
Calculated Nutrient Composition	%
Crude protein	21.44
Crude fat	4.55
Crude fiber	2.17
Calcium	0.95
Total phosphorus	0.71
Available phosphorus	0.45
Sodium	0.16
Potassium	0.92
Chloride	0.27
Lysine	1.31
Methionine	0.56
Total sulfur amino acids	0.91
Threonine	0.87
Tryptophan	0.29
Arginine	1.50
Metabolizable Energy, kcal/kg	3050

^1^ The vitamin mix provided the following (per kilogram of diet): thiamin-mononitrate, 2.4 mg; nicotinic acid, 44 mg; riboflavin, 4.4 mg; D-Ca pantothenate, 12 mg; vitamin B12 (cobalamin), 12.0 g; pyridoxine-HCl, 2.7 mg; D-biotin, 0.11 mg; folic acid, 0.55 mg; menadione sodium bisulfate complex, 3.34 mg; choline chloride, 220 mg; cholecalciferol, 1100 IU; trans-retinyl acetate, 2500 IU; all-rac-tocopherol acetate, 11 IU; ethoxyquin, 150 mg. ^2^ The trace mineral mix provides the following (per kilogram of diet): manganese (manganese (ll) sulfate monohydrate), 101 mg; iron (ferrous sulfate heptahydrate), 20 mg; zinc (Zn), 80 mg; copper (copper (ll) sulfate pentahydrate), 3 mg; iodine (ethylenediamine dihydroiodide), 0.75 mg; magnesium (magnesium oxide), 20 mg; selenium (sodium selenite), 0.3 mg.

**Table 2:** Primers used for amplification of bacteria and cytokines.

Primers	Sequence (5′ to 3′)	Length (Base)	Annealing Temperature (°C)	References
*Bacillus*	F: 5′-ACG GTC GCA AGA CTG AAA CT-3′	20	55	This study
	R: 5′-TCG TAA GTC AAC CCG TGA GA-3′	20		
*E. durans*	F: 5′-CCC ATC AGA AGG GGA TAA CA-3′	20	55	This study
	R: 5′-TTA CCT GCT TTC AGA CTG GC-3′	20		
*S. Enteritidis*	F: 5′-GCA GCG GTT ACT ATT GCA GC-3′	20	60	[43]
	R: 5′-CTG TGA CAG GGA CAT TTA GCG-3′	21		
^1^ IL-10	F: 5′-CAT GCT GCT GGG CCT GAA-3′	18	57.5	[44]
	R: 5′-CGT CTC CTT GAT CTG CTT GAT G-3′	22		
^2^ IL-1β	F: 5′-CTA CAC CCG CTC ACA GTC CT-3′	20	57.5	[44]
	R: 5′-TCA CTT TCT GGC TGGAGG AG-3′	20		
^3^ RPS13	F: 5′-CAA GAA GGC TGT TGC TGT TCG-3′	21	55	[45]
	R: 5′- GGC AGA AGC TGT CGA TGA T-3′	19		
^4^ GAPDH	F: 5′-TCC TGT GAC TTC AAT GGT GA-3′	20	55	[45]
	R: 5′-CAC AAC ACG GTT GCT GTA TC-3′	20		

^1^ IL-10: Interleukin-10; ^2^ IL-1β: Interleukin-1 beta; ^3^ RPS13: ribosomal protein S13; ^4^ GADPH: glyceraldehyde-3-phosphate dehydrogenase.

**Table 3:** Effect of probiotic supplementation and/or *S. Enteritidis* challenge on performance parameters.

	Control		Challenge					
Parameter (d 24)	Basal	Probiotics	Basal	Probiotics	SE	Trt *p* Value	Challenge *p* Value	Trt × Challenge *p* Value
BWG (kg)	1.06	0.97	1.06	1.05	0.05	0.30	0.45	0.41
FCR	1.96	2.05	1.96	1.98	0.07	0.46	0.62	0.60

Birds were randomly assigned to four treatments: basal diet-fed treatment (control), probiotic-fed treatment (probiotic), basal diet-fed and SE-challenged treatment (basal + challenge), or probiotic-fed and SE-challenged treatment (probiotic + challenge). Birds receiving probiotic-fed treatments were supplemented with 0.05% probiotic from the day of hatch through 24 d of age. Birds receiving challenge treatments were orally gavaged with 1 × 10^8^ CFUs/bird *S. Enteritidis* at 14 d of age. Means ± SE; *n* = 6 per treatment group; values with no common superscript within a column differ significantly (*p* < 0.05).

**Table 4:** Effect of probiotic supplementation and/or *S. Enteritidis* challenge on jejunal histological parameters.

	Control		Challenge					
Parameter (d 24)	Basal	Probiotics	Basal	Probiotics	SE	Trt *p* Value	Challenge *p* Value	Trt × Challenge *p* Value
Villus height	97.86	99.14	98.73	92.42	6.48	0.69	0.64	0.55
Crypt depth	17.36	16.48	17.27	19.42	1.23	0.59	0.24	0.22
Villus height/crypt depth	6.10	6.18	5.79	5.11	0.33	0.35	0.05	0.24

Birds were randomly assigned to four treatments: basal diet-fed treatment (control), probiotic-fed treatment (probiotic), basal diet-fed and SE-challenged treatment (basal + challenge), or probiotic-fed and SE-challenged treatment (probiotic + challenge). Birds receiving probiotic treatments were supplemented with 0.05% probiotic from the day of hatching through 24 d of age. Birds receiving challenge treatments were orally gavaged with 1 × 10^8^ CFUs/bird *S. Enteritidis* at 14 d of age. Means; *n* = 6 per treatment group; values with no common superscript within a column differ significantly (*p* < 0.05).

## Data Availability

All relevant data are within the paper.

## Supplementary Materials

The following supporting information can be downloaded at https://www.mdpi.com/article/10.3390/microorganisms13020217/s1. The 16S DNA sequence of *B. subtilis* CE330 (MN848256) and *E. durans* CH33 (MF066896.1).
