# Supplementary material for: In Vitro Characterization of Probiotic Strains Bacillus subtilis and Enterococcus durans and Their Effect on Broiler Chicken Performance and Immune Response During Salmonella Enteritidis Infection
Source: Microorganisms. 2025 Jan 21;13(2):217. doi: 10.3390/microorganisms13020217 (PMC11857266; doi:10.3390/microorganisms13020217)
Supplement: Supplementary file 1 [file microorganisms-13-00217-s001.zip › microorganisms-3405447-supplementary.pdf]

```
LOCUS       MN848256                1371 bp    DNA     linear   BCT 24-DEC-
2019
DEFINITION  Bacillus subtilis strain CE330 16S ribosomal RNA gene, partial
            sequence.
ACCESSION   MN848256
VERSION     MN848256.1
KEYWORDS    .
SOURCE      Bacillus subtilis
  ORGANISM  Bacillus subtilis
            Bacteria; Bacillota; Bacilli; Bacillales; Bacillaceae; Bacillus.
REFERENCE   1  (bases 1 to 1371)
  AUTHORS   Siripornadulsil,W.
  TITLE     Nalisa Khochamit
  JOURNAL   Unpublished
REFERENCE   2  (bases 1 to 1371)
  AUTHORS   Siripornadulsil,W.
  TITLE     Direct Submission
  JOURNAL   Submitted (19-DEC-2019) Microbiology, Khon Kaen University,
            Mittraphap, Khon Kaen 40002, Thailand
COMMENT     ##Assembly-Data-START##
            Sequencing Technology :: Sanger dideoxy sequencing
            ##Assembly-Data-END##
FEATURES             Location/Qualifiers
     source          1..1371
                     /organism="Bacillus subtilis"
                     /mol_type="genomic DNA"
                     /strain="CE330"
                     /isolation_source="Cecum"
                     /host="Broiler"
                     /db_xref="taxon:1423"
                     /country="Thailand"
                     /collection_date="2015"
                     /collected_by="Nalisa Khochamit"
                     /identified_by="Nalisa Khochamit"
     rRNA            <1..>1371
                     /product="16S ribosomal RNA"
ORIGIN
        1 cgagcggaca gatgggagct tgctccctga tgttagcggc ggacgggtga gtaacacgtg
       61 ggtaacctgc ctgtaagact gggataactc cgggaaaccg gggctaatac cggatggttg
      121 tttgaaccgc atggttcaaa cataaaaggt ggcttcggct accacttaca gatggacccg
      181 cggcgcatta gctagttggt gaggtaacgg ctcaccaagg caacgatgcg tagccgacct
      241 gagagggtga tcggccacac tgggactgag acacggccca gactcctacg ggaggcagca
      301 gtagggaatc ttccgcaatg gacgaaagtc tgacggagca acgccgcgtg agtgatgaag
      361 gttttcggat cgtaaagctc tgttgttagg gaagaacaag taccgttcga atagggcggt
      421 accttgacgg tacctaacca gaaagccacg gctaactacg tgccagcagc cgcggtaata
      481 cgtaggtggc aagcgttgtc cggaattatt gggcgtaaag ggctcgcagg cggtttctta
      541 agtctgatgt gaaagccccc ggctcaaccg gggagggtca ttggaaactg gggaacttga
      601 gtgcagaaga ggagagtgga attccacgtg tagcggtgaa atgcgtagag atgtggagga
      661 acaccagtgg cgaaggcgac tctctggtct gtaactgacg ctgaggagcg aaagcgtggg
      721 gagcgaacag gattagatac cctggtagtc cacgccgtaa acgatgagtg ctaagtgtta
      781 gggggtttcc gccccttagt gctgcagcta acgcattaag cactccgcct ggggagtacg
      841 gtcgcaagac tgaaactcaa aggaattgac gggggcccgc acaagcggtg gagcatgtgg
      901 tttaattcga agcaacgcga agaaccttac caggtcttga catcctctga caatcctaga
      961 gataggacgt ccccttcggg ggcagagtga caggtggtgc atggttgtcg tcagctcgtg
     1021 tcgtgagatg ttgggttaag tcccgcaacg agcgcaaccc ttgatcttag ttgccagcat
     1081 tcagttgggc actctaaggt gactgccggt gacaaaccgg aggaaggtgg ggatgacgtc
     1141 aaatcatcat gccccttatg acctgggcta cacacgtgct acaatggaca gaacaaaggg
```

```
     1201 cagcgaaacc gcgaggttaa gccaatccca caaatctgtt ctcagttcgg atcgcagtct
     1261 gcaactcgac tgcgtgaagc tggaatcgct agtaatcgcg gatcagcatg ccgcggtgaa
     1321 tacgttcccg ggccttgtac acaccgcccg tcacaccacg agagtttgta a//
```

```
LOCUS       MF066896                 781 bp    DNA     linear   BCT 13-MAY-
2017
DEFINITION  Enterococcus durans strain CH33 16S ribosomal RNA gene, partial
            sequence.
ACCESSION   MF066896
VERSION     MF066896.1
KEYWORDS    .
SOURCE      Enterococcus durans
  ORGANISM  Enterococcus durans
            Bacteria; Bacillota; Bacilli; Lactobacillales; Enterococcaceae;
            Enterococcus.
REFERENCE   1  (bases 1 to 781)
  AUTHORS   Buahom,J. and Siripornadulsil,W.
  TITLE     Direct Submission
  JOURNAL   Submitted (05-MAY-2017) Microbiology, Khon Kaen University,
            Mittaparp, Khon Kaen 40002, Thailand
COMMENT     ##Assembly-Data-START##
            Sequencing Technology :: Sanger dideoxy sequencing
            ##Assembly-Data-END##
FEATURES             Location/Qualifiers
     source          1..781
                     /organism="Enterococcus durans"
                     /mol_type="genomic DNA"
                     /strain="CH33"
                     /isolation_source="intestine"
                     /host="domestic fowl"
                     /db_xref="taxon:53345"
                     /country="Thailand"
                     /collection_date="11-Jun-2014"
                     /collected_by="Juthamas Buahom"
                     /identified_by="Juthamas Buahom"
                     /note="PCR_primers=fwd_name: 20F, rev_name: 1500R"
     rRNA            <1..>781
                     /product="16S ribosomal RNA"
ORIGIN
        1 taacacgtgg gtaacctgcc catcagaagg ggataacact tggaaacagg tgctaatacc
```

61 gtataacaat cgaaaccgca tggttttgat ttgaaaggcg ctttcgggtg tcgctgatgg
121 atggacccgc ggtgcattag ctagttggtg aggtaacggc tcaccaaggc cacgatgcat
181 agccgacctg agagggtgat cggccacatt gggactgaga cacggcccaa actcctacgg
241 gaggcagcag tagggaatct tcggcaatgg acgaaagtct gaccgagcaa cgccgcgtga
301 gtgaagaagg ttttcggatc gtaaaactct gttgttagag aagaacaagg atgagagtaa
361 ctgttcatcc cttgacggta tctaaccaga aagccacggc taactacgtg ccagcagccg
421 cggtaatacg taggtggcaa gcgttgtccg gatttattgg gcgtaaagcg agcgcaggcg
481 gtttcttaag tctgatgtga aagcccccgg ctcaaccggg gagggtcatt ggaaactggg
541 agacttgagt gcagaagagg agagtggaat tccatgtgta gcggtgaaat gcgtagatat
601 atggaggaac accagtggcg aaggcggctc tctggtctgt aactgacgct gaggctcgaa
661 agcgtgggga gcaaacagga ttagataccc tggtagtcca cgccgtaaac gatgagtgct
721 aagtgttgga gggtttccgc ccttcagtgc tgcagctaac gcattaagca ctccgcctgg
781 g //
